# 3D-GBS: a universal genotyping-by-sequencing approach for genomic selection and other high-throughput low-cost applications in species with small to medium-sized genomes

**DOI:** 10.1186/s13007-023-00990-7

**Published:** 2023-02-05

**Authors:** Maxime de Ronne, Gaétan Légaré, François Belzile, Brian Boyle, Davoud Torkamaneh

**Affiliations:** 1 Département de Phytologie, Université Laval, Quebec, Canada; 2 Institut de Biologie Intégrative et des Systèmes (IBIS), Université Laval, Quebec, Canada; 3 Centre de recherche et d’innovation sur les végétaux (CRIV), Université Laval, Quebec, Canada; 4 Institut intelligence et données (IID), Université Laval, Quebec, Canada

**Keywords:** Genotyping-by-sequencing, Ultra-high-throughput genotyping, Multiplexing, Next-generation sequencing, Genomic selection, Single-nucleotide polymorphism

## Abstract

Despite the increased efficiency of sequencing technologies and the development of reduced-representation sequencing (RRS) approaches allowing high-throughput sequencing (HTS) of multiplexed samples, the per-sample genotyping cost remains the most limiting factor in the context of large-scale studies. For example, in the context of genomic selection (GS), breeders need genome-wide markers to predict the breeding value of large cohorts of progenies, requiring the genotyping of thousands candidates. Here, we introduce 3D-GBS, an optimized GBS procedure, to provide an ultra-high-throughput and ultra-low-cost genotyping solution for species with small to medium-sized genome and illustrate its use in soybean. Using a combination of three restriction enzymes (PstI/NsiI/MspI), the portion of the genome that is captured was reduced fourfold (compared to a “standard” ApeKI-based protocol) while reducing the number of markers by only 40%. By better focusing the sequencing effort on limited set of restriction fragments, fourfold more samples can be genotyped at the same minimal depth of coverage. This GBS protocol also resulted in a lower proportion of missing data and provided a more uniform distribution of SNPs across the genome. Moreover, we investigated the optimal number of reads per sample needed to obtain an adequate number of markers for GS and QTL mapping (500–1000 markers per biparental cross). This optimization allows sequencing costs to be decreased by ~ 92% and ~ 86% for GS and QTL mapping studies, respectively, compared to previously published work. Overall, 3D-GBS represents a unique and affordable solution for applications requiring extremely high-throughput genotyping where cost remains the most limiting factor.

**Supplementary Information:**

The online version contains supplementary material available at 10.1186/s13007-023-00990-7.

## Introduction

Genome-wide genotyping of large populations, an essential component in quantitative trait loci (QTL) mapping or genomic selection (GS) studies, is constantly improving to minimize the cost of genotyping per individual sample. The identification of large numbers of molecular markers has been paralleled by the simultaneous development of high-throughput approaches such as microarray- [18] or sequencing-based genotyping [50]. However, new needs related to applied breeding programs require the development of an ultra-high-throughput and cost-effective genotyping platform. SNP arrays are a popular approach (e.g. BARCSoySNP6K in soybean [55] and C7AIR in rice [42]) providing a robust genotype calling of multiple known polymorphic sites at the same time and across different populations allowing for a direct comparison of data between experiments, germplasm and studies [6, 26]. However, SNP arrays present ascertainment issues [41], an inability to target loci that were not included during the array development and need to be developed independently for each species and population [11]. In addition to these, the cost of genotyping using SNP arrays, even after development, is considerably higher than sequencing-based approaches [15].

While genotyping based on whole-genome sequencing (WGS) remains expensive and sometimes unnecessary in the context of large-scale studies, low- (< 5X) and very low-depth (0.1–0.5X) sequencing approaches, such as skimSeq, have been designed to decrease the sequencing cost for numerous applications in both model and non-model species [37, 58]. However, inferring genotypes from a random sampling of a small percentage of the genome is challenging because very low sequencing coverage often leads to inaccurate genotype calls, particularly for organisms with a high degree of paralogy and or heterozygosity [54]. In contrast, reduced-representation sequencing (RRS) approaches bypass this problem by focusing the sequencing effort on a smaller proportion of the genome that is constant between the genotyped samples (e.g., centered on the exome or on restriction fragments). Combined with high-throughput sequencing (HTS) of multiplexed samples, RRS approaches, allows for a cost-effective genotyping of millions of SNPs in large sets of individuals [23]. Among RRS approaches, genotyping-by-sequencing (GBS) is the most widely used method thanks to its speed, flexibility and cost-effectiveness [21, 43, 47]. In the last decade, GBS has been widely applied in animals [7, 38], plants [2, 72] and fungi [31], where other genotyping tools (e.g., SNP arrays; [18]) were not adapted [8]. The attractiveness of GBS has led to many optimizations related to the choice of enzymes [52], pipeline for calling SNPs [64], improved marker density (double-digest GBS [69] and high-density GBS [65]), and improved library-preparation procedure [62]. Although GBS is the most cost-effective genome-wide genotyping approach, it can still be expensive for routine screening of large populations as required in breeding programs [45, 50, 59]. Nevertheless, GBS could be optimized by focusing sequencing on a lower fraction of the genome allowing more samples to be multiplexed at a lower average sequencing coverage and thus reduce the sequencing cost per sample. Reducing the genome coverage through reduction of sequencing coverage will categorically result in a lower number of markers, however the uniform distribution of these markers is crucial for an efficient and effective genetic study. The appropriate choice of restriction enzymes can also be a challenging point as their recognition sites (based on the size of the enzyme, sensitivity to methylation, and its GC content) are not uniformly distributed across the genomes [24, 34, 39, 44].

The number of required reads is another determining factor in multiplexing and throughput. Despite the various improvements in GBS methods, the estimation of the number of reads for each sample required to achieve an efficient genotyping needs to be determined on a case-by-case basis [66]. An insufficient number of reads per sample will result in a high proportion of missing data, a reduced number of SNP loci at which genotypes can be successfully called and, possibly, an uneven distribution of markers across the genome [14, 25, 60]. In contrast, an excessive number of reads results in an inefficient use of the sequencing effort and therefore, unnecessarily increases per-sample cost [3]. Thus, finding an optimal number of reads per sample can also help minimize per-sample sequencing cost.

To optimize the multiplexing capacity of GBS, a novel combination of three restriction enzymes, hence 3D-GBS, was tested on soybean to reduce the initial number of digested DNA fragments (or sequencing coverage) while producing genotypic data as relevant as stdGBS. The use of this new enzyme combination has improved the distribution of markers across the genome in terms of uniformity and number of gaps compared to stdGBS. Finally, we investigated the optimal number of reads per sample to further maximize multiplexing capacity on a single sequencing run and thereby, significantly minimize the sequencing cost per sample. This approach will greatly facilitate the adoption of ultra-high-throughput genome-wide genotyping where the per-sample cost remains a limiting factor for various applications.

## Materials and methods

### Biological materials

To compare the GBS [15] and 3D-GBS methods, sixteen soybean accessions (QS4049, QS4054, QS4067, QS5008, QS4028, QS4043, QS5017, OAC Klondike, OAC Bright, Altesse, OAC Inwood, OAC Thames, OAC 08-18C, OAC Morris, OAC Embro and OAC McCall; provided by Dr. Louise O’Donoughue at CEROM, Quebec, QC, Canada) were used in this study. These accessions were selected based on the availability of GBS data [53]. For each accession, seeds were grown in a growth chamber. Then, approximately 100 mg of young leaf tissues were collected for DNA extraction. Collected leaf tissues were dried for 4 days using a desiccating agent (Drierite; Xenia, OH, USA) and then ground with metallic beads in a RETSCH MM 400 mixer mill (Fisher Scientific, MA, USA). DNA was extracted using the DNeasy Plant Mini Kit (Qiagen, MD, USA) according to the manufacturer’s protocol. DNA quantification was done with a Qubit fluorometer using the dsDNA HS assay kit (Thermo Fisher Scientific, MA, USA) and subsequently adjusted to 10 ng/µl for each sample.

### 3D-GBS library preparation

#### Choice of enzymes

The restriction enzymes for 3D-GBS were selected based on their sensitivity to methylation and the size of their recognition site compared to ApeKI, a standard GBS protocol for soybean. ApeKI is a 5 bp-cutter with one ambiguous site and 80% GC content (G*CWGC). Here, we used following enzymes: PstI, a 6-bp cutter with 66% GC content (CTGCA*G), NsiI, a 6-bp cutter with 33% GC content (ATGCA*T), and MspI, a 4-bp cutter with 100% GC content (C*CGG). *Ape*KI and *Pst*I are partially sensitive and sensitive to cytosine methylation, respectively, while NsiI and MspI are not sensitive to cytosine methylation.

#### Library preparation

3D-GBS libraries were prepared on a reduced scale (5 µL reaction volume) according to the NanoGBS protocol [62] with the three selected enzymes (PstI, NsiI and MspI). Briefly, a total of 10 ng of genomic DNA of each sample was used for digestion with the restriction-enzyme mix and then ligation with sample-specific barcoded adapters. The 5’ adapters had an overhang compatible with the common overhang produced by PstI and NsiI, while the 3′ adapters had an overhang compatible with that produced by MspI. Then, individual libraries were pooled and a size-selection (50–350 bp) step was done using a BluePippin apparatus (Sage Science, MA, USA). PCR amplification (12 cycles), enrichment, and PCR clean-up were performed before quality control, quantification, and purity assessments of DNA libraries with a spectrophotometer (Nanodrop 1000, Fisher Scientific, MA, USA) and a Bioanalyzer 2100 (Agilent Technologies, CA, USA). The 3D-GBS libraries were then sequenced on an Ion Torrent instrument (Thermo Fisher Scientific, MA, USA) on Ion Proton 540 chips at the Genomic Analysis Platform of the Institut de Biologie Intégrative et des Systèmes (Université Laval, QC, Canada).

### Data analysis

#### Sequencing and genotyping

Sequencing data were processed using the Fast-GBS v2.0 pipeline [64] and the Wm82.a2 soybean reference genome (Gmax_275_Wm82.a2.v1, [51]) for SNP calling. For GBS and 3D-GBS analyses, variant calls were filtered with VCFtools [9] to remove low-quality SNPs (QUAL < 10 and MQ < 30), variants residing on unassembled scaffolds and indels. Then, only biallelic markers with missing data < 0.8 and heterozygosity < 0.1 were retained. This filtering step resulted in the removal of approximately 70% of low-quality variants from both GBS (25,280 to 7904) and 3D-GBS (15,082 to 4826) data. The following statistical analysis were performed using filtered data. The genome coverage (fraction of the genome captured) was determined with the function ‘coverage’ in Samtools [10] while the mean depth of coverage (sequencing coverage) was calculated using VCFtools with the function ‘–depth’. The proportion of missing data and heterozygous calls, average minor allele frequency and nucleotide diversity (PiPerBP) were estimated using TASSEL v.5 [5]. In silico digestion analysis was performed using DepthFinder [66] to determinate the number of cutting site across the genome for different combinations of enzymes.

#### Distribution of markers on the physical and genetic maps

The distribution of markers across the physical map was based on the VCF files generated after Fast-GBS analysis and SNP filtration, using the rMVP package in R [70]. For genetic maps, the genetic position of each SNP was inferred from the closest corresponding SNP on the consensus genetic map based on GBS-derived SNP markers [16]. Then, the distribution of markers across the genetic maps was evaluated using the QTL IciMapping v4.1 software [40].

#### Random sampling of reads

Different subsets of reads (i.e., 50K, 100K, 200K and 300K reads) were randomly sampled three times for each of the 16 accessions using seqtk [32] with the function ‘sample’ [32]. Then, sequencing data as well as the number and distribution of SNPs were assessed as mentioned above to compare results generated from each read subgroup. To investigate 3D-GBS results for biparental crosses, the two genetically closest and most distant accessions were determined by using a matrix of pairwise distances generated with TASSEL v.5 [49].

## Results and discussion

### New enzyme combinations for an efficient and uniform capture of the genome

**Fig. 1:**
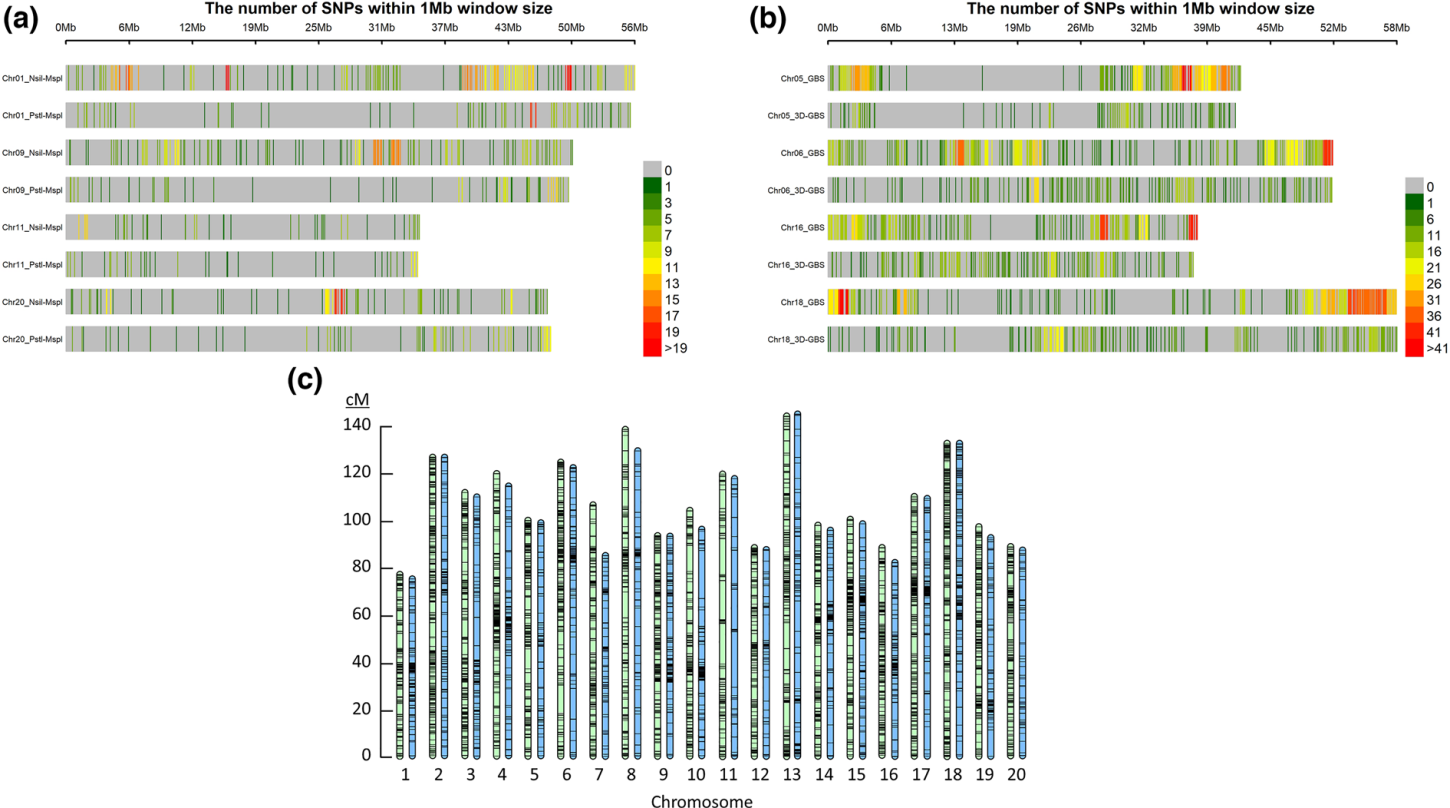
Distribution of the GBS- and 3D-GBS-derived SNPs across the soybean genome. **a** Distribution of the SNPs derived from NsiI–MspI and PstI–MspI reads on selected chromosomes. The colors of the heatmap correspond to the number of SNPs within 1-Mb windows. **b** Distribution of the SNPs derived from GBS and 3D-GBS on selected chromosomes. **c** Distribution of the SNPs derived from GBS and 3D-GBS libraries across the soybean genetic map. Chromosomes in green and blue represent GBS and 3D-GBS, respectively

In this study, sixteen DNA samples that had been previously genotyped with the original ApeKI-based GBS protocol were used to produced 3D-GBS libraries. The 16-plex GBS and 3D-GBS libraires produced ~ 21.1M (ranging from ~ 800K to ~ 2.9M reads/sample) and ~ 10.4M (ranging from ~ 300K to ~ 800K reads/sample) reads, respectively. First, the distribution of the SNPs derived from PstI–MspI and Nsil–MspI reads was investigated to assess the relevance of this enzyme combination (Fig. 1a and Additional file 1: Fig. S1). We found 76.5% and 23.5% of Nsil–MspI and PstI–MspI reads, respectively, encompassing 4206 and 620 SNPs, respectively. The higher proportion of NsiI–MspI-derived fragments and SNPs could be expected because of the methylation insensitivity of NsiI and lower GC content compared to PstI. Nevertheless, PstI–MspI-derived fragments ensured the coverage of large gaps devoid of NsiI–MspI-derived fragments (e.g., on chromosomes 9, 11 and 20).

To perform a meaningful comparison, the same overall number of reads for each 16-plex library was used to compare the two protocols; as the number of reads per accession varied, an identical number of mapped reads for a given accession in each of the two libraries was used to compare GBS and 3D-GBS (Table 1). As expected, with 3D-GBS, a lower fraction of the genome was captured compared to GBS (genome coverage of 1.2% vs 4.7%, respectively). As the sequencing effort (i.e., the number of reads per sample) was focused on a smaller fraction of the genome, the mean depth of coverage was threefold higher in 3D-GBS compared to GBS (14.5X vs 5.1X, respectively) resulting in a lower proportion of missing data (15.3% vs 33.7%, respectively). Fortunately, while the genome coverage was 75% lower for 3D-GBS than GBS data, the number of SNPs identified was only 40% lower (4826 vs 7904 SNPs, respectively), showing that 3D-GBS either captures more polymorphic regions of the genome or improves the genotyping efficiency for the same sequencing effort. As expected, highly similar metrics were obtained for mapping quality, proportion of heterozygous genotypes, average minor allele frequency and nucleotide diversity in both datasets. This suggests that 3D-GBS data is as relevant as GBS data for performing different genetic analyses.

**Table 1:** Sequencing and SNP-calling data generated from GBS and 3D-GBS libraries of 16 soybean samples

Steps	Measured parameters	GBS	3D-GBS
Sequencing	Mean read count (M)	0.6	0.6
	Coverage (%)^a^	4.7	1.2
	Mean depth of coverage (X)^b^	5.1	14.5
	Mean mapping quality	41	42
SNP calling	SNP count	7904	4826
	Proportion of missing data (%)	33.7	15.3
	Proportion of heterozygous genotypes (%)	4.4	3.8
	Average minor allele frequency (%)	33.8	31.3
	Nucleotide diversity (p per bp)	0.43	0.42
Physical map	SNP/Mb	8.3	5.1
	Number of gaps > 5 Mb	9	6
	Number of gaps > 10 Mb	1	0
Genetic map	SNP/cM^c^	3.7	2.3
	Number of gaps > 10 cM	7	10
	Number of gaps > 20 cM	0	1

^a^Fraction of the genome captured across all 16 libraries

^b^Average number of read at each sequenced position

^c^Inferred from the closest corresponding SNP on the consensus genetic map [16]

The density of SNPs captured by 3D-GBS (5.1 SNPs/Mb and 2.3 SNPs/cM with no gap > 30 cM) represents an adequate density to perform QTL mapping and GS analysis. To confirm this, the distribution of the SNPs across the physical and genetic maps has been evaluated (Fig. 1b, c, Additional file 2: Fig. S2). Compared to GBS-derived SNPs, the distribution of the 3D-GBS-derived SNPs was more uniform across the genome (Fig. 1b and Additional file 2: Fig. S2). This can be easily illustrated by (i) several regions > 5 Mb on chromosomes 1, 5, 6, 12, 16 and 18 that are missed by GBS while they were covered by 3D-GBS; and (ii) the more uniform distribution of SNPs which rarely exceeds 25 SNPs/Mb in 3D-GBS, compared to GBS where many regions are covered with an “excessive” number of SNPs (25 to more than 41 SNPs/Mb; e.g. on chromosomes 4, 5, 6, 16, 18, etc.). Finally, regarding the genetic map, the 3D-GBS SNPs were well distributed with only one gap close to 20 cM on Chr11, in a region that was also poor in GBS-derived SNPs (Fig. 1c), suggesting that 3D-GBS data are as efficient as GBS data to conduct genetic analyses such as GS or QTL mapping.

The appropriate choice of enzyme(s) is an essential step in developing a GBS protocol [20]. In the original GBS protocol [15], the ApeKI enzyme was used as frequent cutter with sensitivity to methylation to obtain SNPs mainly distributed in gene-rich regions (hypomethylated fraction of the genome) corresponding to a coverage of ~ 4–5% of the genome (Table 1). A two-enzyme strategy using a rare (e.g. PstI) and a frequent cutter (e.g. MspI) sensitive to methylation has also been developed to significantly reduce genome complexity in species with a very large genome (e.g., barley (5 Gb) [46]). However, this approach did not show enough efficiency with species with small to medium genome size [e.g., soybean (⁓1 Gb)] as it captured relatively few genomic regions [65]. Moreover, due to the palindromic nature of enzyme’s restriction sites, this produces a bias in GC content, making the two-enzyme strategy using a rare cutter (none available with 50% GC content) impossible to obtain uniform distribution of fragments in the context of a universal use. Indeed, since there is natural variation in GC content across chromosomes [24, 34, 39, 44] and between species [29, 35], using a rare cutter with either 33% or 66% of GC will inevitably induce variable density of restriction fragments across chromosomes and species. On the other hand, frequent cutters can have a 50% GC content, such as MspI (CCGG) or BfaI (CTAG), allowing a more even distribution of restriction fragments throughout the genome, as illustrated by Torkamaneh et al. [65]. However, when they have been used alone, these frequent cutters induce too many restriction fragments across the genome, which is contrary to the objective of reducing genome coverage.

In light of the above, we explored the idea of improving the two-enzyme approach by using a second rare cutter, such as NsiI [17], with a cutting site differing in GC content and exploiting methylation insensitivity to capture hypermethylated regions missed by PstI. The combination of NsiI with PstI and MspI presented a good opportunity to obtain a sufficient and efficient low density of SNPs distributed more evenly in the genome. While ApeKI would be expected to cut every ~ 512 bp (4^4.5^), here, a combination of three enzymes that include PstI and NsiI (two 6-bp-cutter with differing methylation sensitivity), with a predicted cutting frequency of one site every ~ 4096 bp (4^6^), and MspI, a methylation-insensitive 4-bp cutter with an expected cutting frequency of one site every 256 bp (4^4^) were used jointly to reduce the fraction of the genome that is captured. The high cutting frequency of MspI allows to generate more fragments of 100–400 bp [22] that are ideal for short-read sequencing. Together, these enzymes span a broad GC, 33% for NsiI, 66% for PstI and 100% for MspI, thus creating a suitable condition to reduce genome coverage and uniformly sample different genomic regions. Based on in silico digestion analysis, different combination of enzymes with similar cutting site criteria (size and GC content) could be considered to further reduce genome coverage (Additional file 3: Fig. S3). Finally, by focusing on fewer but well-distributed genomic regions, 3D-GBS offers an efficient and cost-effective approach for discovery and genotyping of SNPs across the genome in species with small to medium-sized genome.

### Optimizing the number of reads per sample to maximize multiplexing

Different numbers of reads (i.e. 50K, 100K, 200K and 300K reads) were randomly sampled three times for each accession from the 16-plex 3D-GBS library. For each metric investigated, the coefficient of variation between replicates based on the same number of reads was < 5% [not significantly different (Tukey HSD test *p*-value > 0.1)]. For this reason, the mean value (across all three replicates) for each metric is reported in Table 2. With increasing the sequencing effort from 50 to 300K reads per sample, the fraction of the genome captured increased from 0.6 to 1%, the number of SNPs increased from 1314 to 4082, and the proportion of missing data decreased from 37 to 20%. Even at the smallest value tested (50K reads/sample), the proportion of missing data was still reasonable and would allow for an accurate imputation [61]. For average minor allele frequency and nucleotide diversity values, equivalent results were provided across the entire range of reads per sample, suggesting that even with a very limited sequencing effort one can perform high-quality genetic diversity analysis.

**Table 2:** Variant calling using different subsets of reads derived from 3D-GBS on 16 soybean samples

Step	Measured parameters	50K reads	100K reads	200K reads	300K reads
Sequencing	Coverage (%)^a^	0.6	0.7	0.9	1
	Mean depth of coverage (X)^b^	2.7	4.1	6.3	8.4
SNP calling	SNP count	1,314	2,299	3,587	4,082
	Proportion of missing data (%)	37.1	29.3	23.3	20.3
	Proportion of heterozygous genotypes (%)	6.1	5.2	5	4.6
	Average minor allele frequency (%)	27.3	27.2	26.2	25.9
	Nucleotide diversity (p per bp)	0.36	0.36	0.35	0.35
Physical map	SNP/Mb	1.4	2.4	3.8	4.3
	Number of gaps > 5 Mb	23	9	5	7
	Number of gaps > 10 Mb	6	1	1	0
Genetic map	SNP/cM^c^	0.6	1.1	1.7	2
	Number of gaps > 10 cM	29	18	12	9
	Number of gaps > 20 cM	2	1	1	1

^a^Total genome fraction captured by the 16 libraries

^b^Average number of read at each sequenced position

^c^Inferred from the closest corresponding SNP on the consensus genetic map [16]

**Fig. 2:**
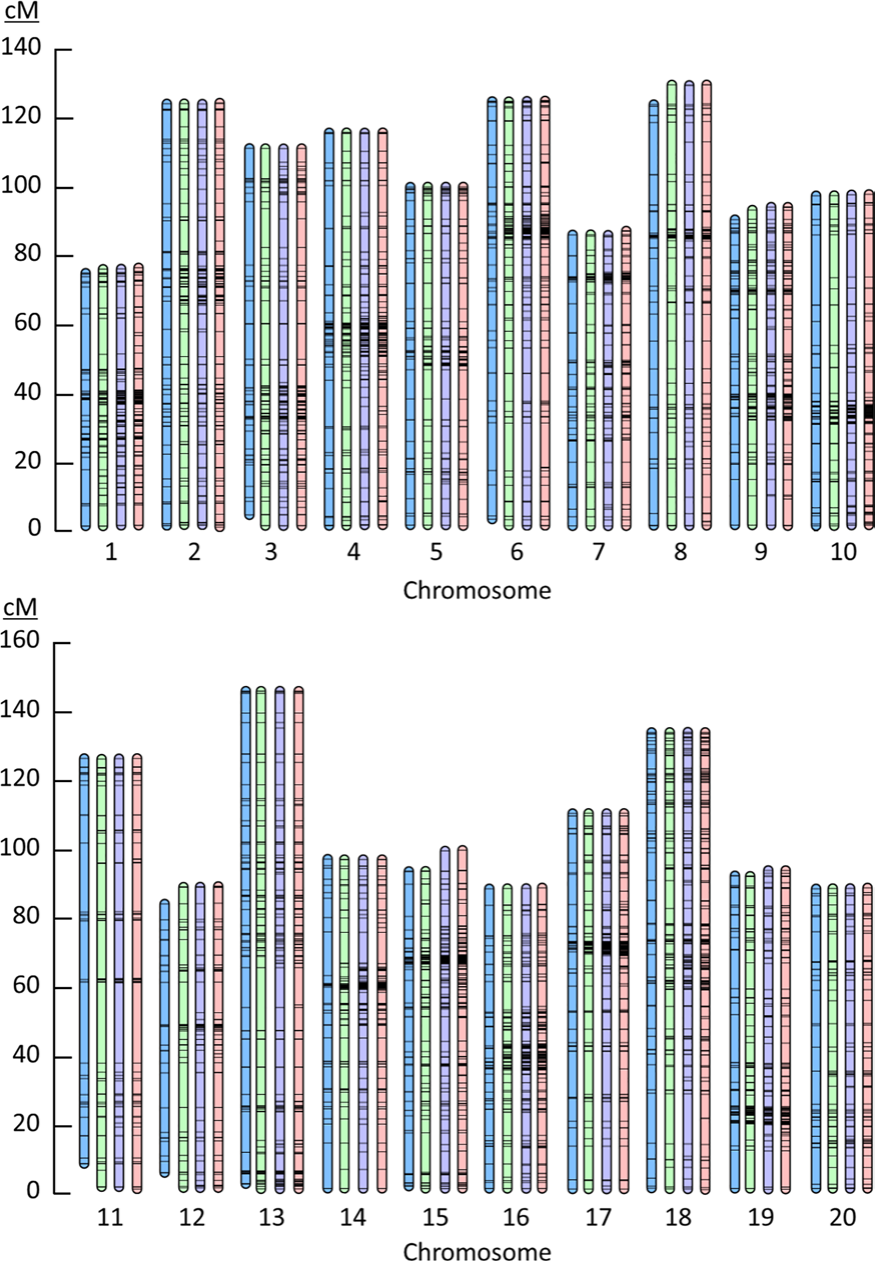
Comparison between genetic maps based on different number of 3D-GBS reads. Genetic map in blue, green, purple and red were constructed based on 50K, 100K, 200K and 300K reads, respectively

The distribution of the SNPs on the genetic map was very similar from 100 to 300K reads while, with only 50K reads per sample, large gaps were detected (e.g., ~ 10 cM on Chr01, ~ 80 cM on Chr03, ~ 60 cM on Chr06) and some chromosome extremities were missed (Fig. 2). While the density of markers doubled between 100 and 300K reads, the distribution of the SNPs across the genetic map remained very similar with some regions that were denser in SNPs using 300K reads (e.g., ~ 40 cM on Chr01, ~ 60 cM on Chr04, ~ 90 cM on Chr06). This very promising result suggests that one can run 3D-GBS with only 100K reads per sample, a significant reduction in the sequencing cost, to achieve sufficient resolution (~ 2300 SNPs, 1.1 SNP/cM) to perform GS.

In the case of mapping studies using biparental populations (i.e. QTL mapping), the number of polymorphic marker loci can significantly vary based on the relatedness of parents. To ensure that the proposed number of reads would still offer a sufficient number of markers for biparental QTL mapping, we determined the number and distribution of SNPs between the least and most genetically distant pairs of accessions within this collection. A matrix of pairwise genetic distance among the 16 accessions was produced and identified QS4054 and OAC Bright as the most genetically similar, while QS5008 and QS4067 proved to be the most distant (Additional file 4: Table S1). The number of polymorphic markers using 100K and 300K reads varied from 426 to 669 for the closest lines and from 677 to 1325 for the most distant ones (Table 3). This means that for the closest lines, doubling or tripling the number of reads from 100K reads had only allowed the discovery of 32% and 36% more SNPs, respectively. In contrast, in the most distant lines, doubling or tripling of the number of reads from 100K has doubled the density of markers on the genetic map. Thus, as similar results were obtained between 200 and 300K, 200K reads per sample seems as suitable as 300K reads to perform QTL mapping in a biparental population. This represents a significant gain compared to current studies where ApeKI-based GBS protocol was used with over 1M reads per sample to conduct QTL mapping studies [12, 57].

**Table 3:** Analysis of SNP density obtained with different number of reads for two hypothetical biparental crosses

Crossing	Closest accessions	Farthest accessions	Closest accessions	Farthest accessions	Closest accessions	Farthest accessions
Reads per sample (K)	100		200		300	
SNP count	426	677	630	1165	669	1325
SNP/1 Mb	0.5	0.7	0.7	1.2	0.7	1.4
SNP/cM	0.21	0.33	0.31	0.57	0.33	0.65

The genetically closest and farthest accessions were QS4054 and OAC Bright and, QS5008 and QS4067, respectively

Compared to other low- (< 5X) and very low-depth (0.1–0.5X) sequencing approaches developed to reduce the cost of genotyping [37, 58], 3D-GBS can be considered as extremely low-depth sequencing (~ 0.01X). Low-depth sequencing methods suffer from genotype uncertainty as a limited amount of sequencing reads are normally used to cover the entire genome. As an example, 1M reads (100 bp) provide a mean depth of coverage of 0.1X of a medium size genome (e.g., soybean; 1 Gb). In 3D-GBS, 200K reads provide a mean depth of coverage of 6X as the complexity reduction allows to focus the sequencing effort on a small proportion of the genome. Furthermore, 3D-GBS offers markers that are better distributed across the genome. Finally, here 3D-GBS libraries were prepared with the least expensive NGS library preparation procedure and its data can also be processed with efficient and user-friendly bioinformatic pipelines [62, 64].

### Maximizing multiplexing to minimize the sequencing cost per sample

Thanks to its efficiency and low cost, the GBS approach is commonly used to perform genome-wide genotyping for a large number of species (animal [19], plant [4], insect [13] and microorganism [31]) and different applications (association studies [63] and GS [27, 48]). Nevertheless, the cost associated with high-throughput screening for genome-wide markers remains the most limiting factor in the context of large-scale studies such as GS, genetic fingerprinting and genetic diversity studies. In association studies (GWAS), in general, the denser the catalog of SNPs, the higher the mapping resolution will be. However in contrast, in most of genetic studies (e.g., GS), linkage disequilibrium (LD) is very extensive and a low density SNP catalog is sufficient to capture linkage blocks and perform the analysis [49, 68]. Recent studies based on reducing the total number of SNPs by focusing on a subset with significant marker-trait associations [33, 56] or based on functional annotations [30], suggest that a lower-density catalog could generate prediction accuracies as high or better than dense catalogs (e.g., WGS-based genotyping) [36]. This has been well illustrated for GS in barley, where Abed et al. [1] showed that a catalog of 2K GBS-SNPs provided a very similar prediction accuracy compared to 35K SNPs.

As documented before [62], to reduce the genotyping cost, one can decide to increase the multiplexing level by decreasing the sequencing effort per sample, which can, however, lead to a higher proportion of missing data that need to be imputed correctly and a non-uniform distribution of SNPs across the genome [63, 64]. Here, using 3D-GBS, we showed that it is possible to produce a lower number of restriction fragments, well and uniformly distributed across the genome, to reduce the number of reads needed to provide sufficient read coverage to call genotypes efficiently. Here, we found that 100K reads is sufficient to conduct GS with 3D-GBS, and that is significantly lower compared to previous studies where GBS has been used (e.g., Qin et al. [48] with ~ 3.3M reads/sample, Jarquín et al. [27] with ~ 2.6M reads/sample and Jean et al. [28] with ~ 1.2M reads/sample). Similarly, we estimated the optimal number of reads per sample for an efficient genotyping of bi- and multi-parent populations. In the context of biparental populations, we estimated that 200K reads/sample is suitable for performing QTL mapping. 3D-GBS allowed a drastic reduction compared to equivalent studies using GBS where a much larger number of reads per sample were used (e.g., Yoon et al. [71] ~ 3.2M, Heim and Gillman [22] ~ 2.4M, St-Amour et al. [57] ~ 1.4M, de Ronne et al. [12] ~ 1.0M and Vuong et al. [67] ~ 843K).

To estimate the gain of 3D-GBS over the standard GBS approach, we selected two studies conducted internally, using ApeKI-based GBS protocol and with the lowest number of reads per sample for GS [28] and QTL mapping [12]. In these study cases, based on the optimal number of reads/sample estimated previously, with the same population, experimental design and goal, the application of 3D-GBS for GS and QTL mapping would have led to similar results with a significant reduction in per-sample sequencing cost: ~ 92% (~ 1.2M vs 100K reads/sample) and ~ 86% (~ 1.4M vs 200K reads/sample), respectively. All without taking into account the miniaturization of sequencing libraries which alone can reduce library preparation costs by 67% [62]. Overall, the combination of recent improvements in miniaturizing GBS library preparation procedure (i.e., NanoGBS [23]) and 3D-GBS provides a unique opportunity to dramatically reduce per-sample genotyping costs.

## Conclusion

Recent advances in NGS technologies have enabled the massively parallel processing of hundreds of samples efficiently and cost-effectively, a prerequisite for genetic studies such as QTL mapping and GS. However, it still remains costly in the context of large-scale studies such as GS, as breeding programs typically produce many thousands of selection candidates each year. In the continuous objective of reducing the genotyping cost for scientific research and applied needs, 3D-GBS enables us to maximize the multiplexing capacity needed to achieve the ultra-high throughput that is needed in a wide range of applications and thus decreasing the sequencing cost per sample. While we demonstrated the efficiency of 3D-GBS using soybean samples, this method could easily be used across a wide range of species with small to medium genome size.

## Supplementary Information

**Additional file 1: Figure S1.** Distribution of the SNPs derived from NsiI–MspI and PstI–MspI reads across the physical map. The colors of the heatmap correspond to the number of SNPs within 1 Mb windows size.

**Additional file 2: Figure S2.** Distribution of the SNPs derived from GBS and 3D-GBS libraries across the physical map. The colors of the heatmap correspond to the number of SNPs within 1 Mb windows size.

**Additional file 3: Figure S3.** Predicted number of cutting site derived from in silico digestion with different restriction enzyme. These enzyme span a GC content of 33% for BglII, BclI and NsiI, 66% for BlnI, BamHI and PstI, and 100% for BfaI, BstUI and MspI. Each color represents a combination of different enzyme.

**Additional file 4: Table S1.** Heatmap of pairwise genetic distance between the 16 soybean accessions. Green to red reflect low to high genetic distance (smallest and largest values are 0.26 and 0.56, respectively).

## Data Availability

The VCF files generated from the sequencing data and used for the analyzes of this study are on FigShare.com and will be accessible after acceptance of the manuscript.

## Abbreviations

NGS: Next-generation sequencing
HTS: High-throughput sequencing
SNP: Single-nucleotide polymorphism
QTL: Quantitative trait loci
GWAS: Genome-wide association study
MAS: Marker-assisted selection
GS: Genomic selection
WGS: Whole-genome sequencing
RRS: Reduced-representation sequencing
RAD: Restriction-associated DNA
CRoPS: Complexity reduction of polymorphic sequences
GBS: Genotyping-by-sequencing
LD: Linkage disequilibrium
