# Supplementary figures and images for: 3D-GBS: a universal genotyping-by-sequencing approach for genomic selection and other high-throughput low-cost applications in species with small to medium-sized genomes
Source: Plant Methods. 2023 Feb 5;19:13. doi: 10.1186/s13007-023-00990-7 (PMC9899395; doi:10.1186/s13007-023-00990-7)

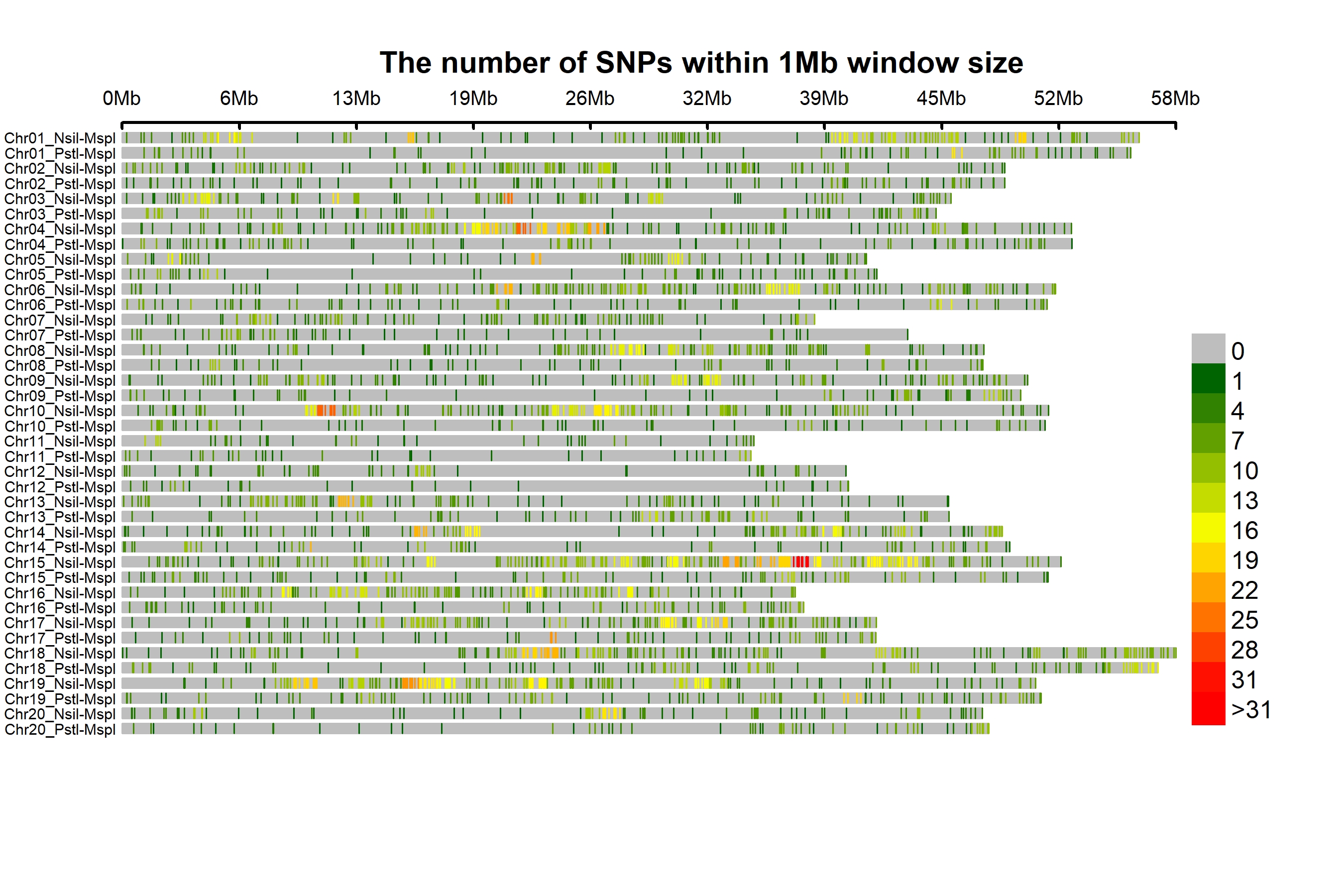

Supplement: Supplementary file 1 — Additional file 1: Figure S1. Distribution of the SNPs derived from NsiI–MspI and PstI–MspI reads across the physical map. The colors of the heatmap correspond to the number of SNPs within 1 Mb windows size. [file 13007_2023_990_MOESM1_ESM.tiff]

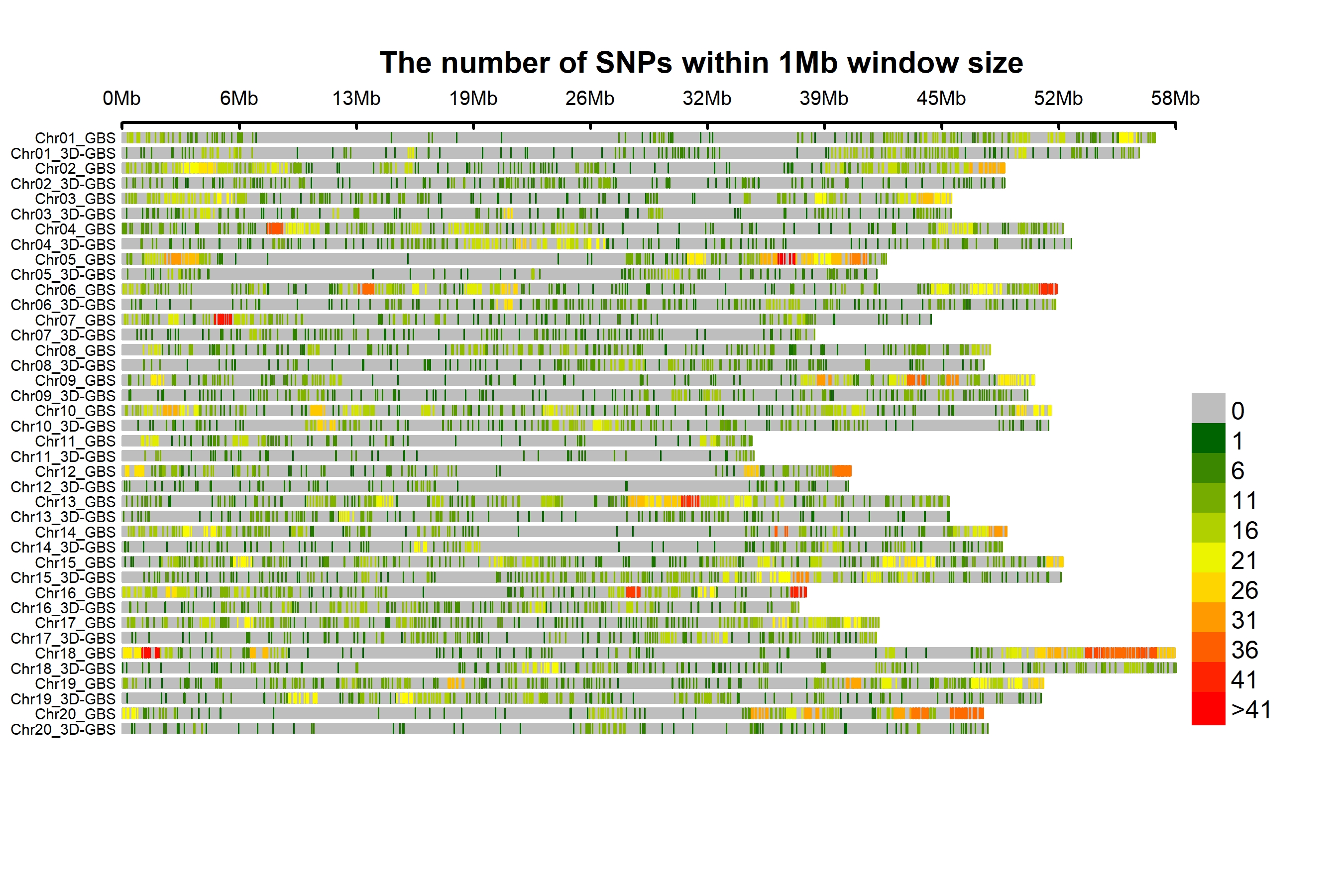

Supplement: Supplementary file 2 — Additional file 2: Figure S2. Distribution of the SNPs derived from GBS and 3D-GBS libraries across the physical map. The colors of the heatmap correspond to the number of SNPs within 1 Mb windows size. [file 13007_2023_990_MOESM2_ESM.tiff]

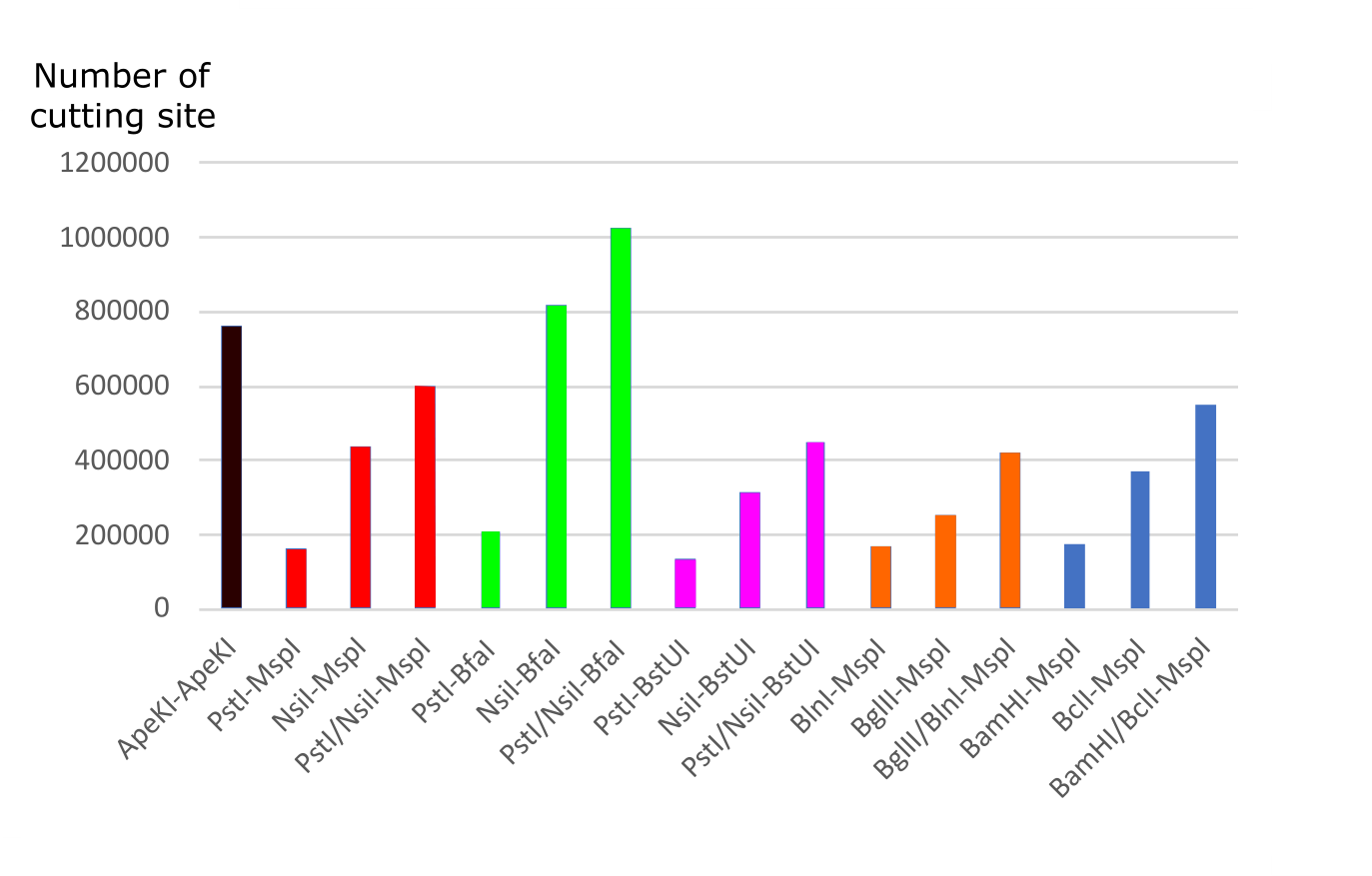

Supplement: Supplementary file 3 — Additional file 3: Figure S3. Predicted number of cutting site derived from in silico digestion with different restriction enzyme. These enzyme span a GC content of 33% for BglII, BclI and NsiI, 66% for BlnI, BamHI and PstI, and 100% for BfaI, BstUI and MspI. Each color represents a combination of different enzyme. [file 13007_2023_990_MOESM3_ESM.tiff]
