# Supplementary material for: 3D-GBS: a universal genotyping-by-sequencing approach for genomic selection and other high-throughput low-cost applications in species with small to medium-sized genomes
Source: Plant Methods. 2023 Feb 5;19:13. doi: 10.1186/s13007-023-00990-7 (PMC9899395; doi:10.1186/s13007-023-00990-7)
Supplement: Supplementary file 4 — Additional file 4: Table S1. Heatmap of pairwise genetic distance between the 16 soybean accessions. Green to red reflect low to high genetic distance (smallest and largest values are 0.26 and 0.56, respectively). [file 13007_2023_990_MOESM4_ESM.docx]

|  | **Altesse** | **McCall** | **OAC 08-18C** | **OAC Bright** | **OAC Embro** | **OAC Inwood** | **OAC Klondike** | **OAC Morris** | **OAC Thames** | **QS4028** | **QS4043** | **QS4049** | **QS4054** | **QS5017** | **QS4067** | **QS5008** |
| --- | --- | --- | --- | --- | --- | --- | --- | --- | --- | --- | --- | --- | --- | --- | --- | --- |
| **Altesse** | 0 | 0.44 | 0.39 | 0.37 | 0.47 | 0.36 | 0.43 | 0.49 | 0.33 | 0.43 | 0.34 | 0.49 | 0.38 | 0.39 | 0.47 | 0.40 |
| **McCall** | 0.44 | 0 | 0.47 | 0.44 | 0.44 | 0.40 | 0.35 | 0.48 | 0.37 | 0.48 | 0.33 | 0.48 | 0.45 | 0.48 | 0.45 | 0.43 |
| **OAC 08-18C** | 0.39 | 0.47 | 0 | 0.40 | 0.40 | 0.42 | 0.47 | 0.49 | 0.46 | 0.43 | 0.45 | 0.38 | 0.41 | 0.50 | 0.44 | 0.44 |
| **OAC Bright** | 0.37 | 0.44 | 0.40 | 0 | 0.33 | 0.31 | 0.46 | 0.46 | 0.37 | 0.39 | 0.32 | 0.39 | **0.26** | 0.51 | 0.46 | 0.35 |
| **OAC Embro** | 0.47 | 0.44 | 0.40 | 0.33 | 0 | 0.43 | 0.43 | 0.43 | 0.36 | 0.37 | 0.43 | 0.39 | 0.34 | 0.53 | 0.47 | 0.45 |
| **OAC Inwood** | 0.36 | 0.40 | 0.42 | 0.31 | 0.43 | 0 | 0.46 | 0.47 | 0.41 | 0.44 | 0.37 | 0.40 | 0.34 | 0.49 | 0.51 | 0.40 |
| **OAC Klondike** | 0.43 | 0.35 | 0.47 | 0.46 | 0.43 | 0.46 | 0 | 0.46 | 0.36 | 0.43 | 0.34 | 0.47 | 0.37 | 0.49 | 0.39 | 0.43 |
| **OAC Morris** | 0.49 | 0.48 | 0.49 | 0.46 | 0.43 | 0.47 | 0.46 | 0 | 0.43 | 0.43 | 0.47 | 0.48 | 0.45 | 0.36 | 0.35 | 0.53 |
| **OAC Thames** | 0.33 | 0.37 | 0.46 | 0.37 | 0.36 | 0.41 | 0.36 | 0.43 | 0 | 0.40 | 0.30 | 0.43 | 0.34 | 0.47 | 0.43 | 0.46 |
| **QS4028** | 0.43 | 0.48 | 0.43 | 0.39 | 0.37 | 0.44 | 0.43 | 0.43 | 0.40 | 0 | 0.46 | 0.35 | 0.37 | 0.46 | 0.44 | 0.35 |
| **QS4043** | 0.34 | 0.33 | 0.45 | 0.32 | 0.43 | 0.37 | 0.34 | 0.47 | 0.30 | 0.46 | 0 | 0.49 | 0.30 | 0.46 | 0.47 | 0.33 |
| **QS4049** | 0.49 | 0.48 | 0.38 | 0.39 | 0.39 | 0.40 | 0.47 | 0.48 | 0.43 | 0.35 | 0.49 | 0 | 0.38 | 0.50 | 0.45 | 0.40 |
| **QS4054** | 0.38 | 0.45 | 0.41 | **0.26** | 0.34 | 0.34 | 0.37 | 0.45 | 0.34 | 0.37 | 0.30 | 0.38 | 0 | 0.52 | 0.49 | 0.34 |
| **QS5017** | 0.39 | 0.48 | 0.50 | 0.51 | 0.53 | 0.49 | 0.49 | 0.36 | 0.47 | 0.46 | 0.46 | 0.50 | 0.52 | 0 | 0.47 | 0.44 |
| **QS4067** | 0.47 | 0.45 | 0.44 | 0.46 | 0.47 | 0.51 | 0.39 | 0.35 | 0.43 | 0.44 | 0.47 | 0.45 | 0.49 | 0.47 | 0 | **0.56** |
| **QS5008** | 0.40 | 0.43 | 0.44 | 0.35 | 0.45 | 0.40 | 0.43 | 0.53 | 0.46 | 0.35 | 0.33 | 0.40 | 0.34 | 0.44 | **0.56** | 0 |

**Table S1**: Heatmap of pairwise genetic distance between the 16 soybean accessions. Green to red reflect low to high genetic distance (smallest and largest values are 0.26 and 0.56, respectively).
